# Perspectives on the impact of the inclusion of non-medical practitioners in the skill mixed staffing teams of English emergency departments: a qualitative study

**DOI:** 10.1186/s12913-026-14832-4

**Published:** 2026-05-29

**Authors:** Vari M. Drennan, Francesca Taylor, Mary Halter, Jonathan Gabe, Heather Gage, Heather Jarman, Sakshi Adhav

**Affiliations:** 1 Faculty of Health, Science, Social Care and Education, Kingston University, London, KT2 7LF UK; 2 The School of Law and Social Sciences, Royal Holloway, University of London, Egham, Surrey, TW20 0EX UK; 3 Surrey Health Economics Centre, Faculty of Health and Medical Sciences, University of Surrey, Guildford, Surrey, GU2 7XH UK; 4 St George’s University Hospitals NHS Foundation Trust, London, SW17 0QT UK

**Keywords:** Health workforce, Emergency department, Skill-mix, Non-medical practitioners, Advanced clinical practitioners, Physician assistants/associates, Qualitative interviews

## Abstract

**Background:**

Emergency Departments internationally have struggled to meet increased patient demand in the context of staffing shortages, particularly doctors. Some countries have employed non-medical practitioners (NMPs), such as advanced nurse practitioners and physician assistants, who undertake some of the clinical work of the doctors across the range of patients attending, not just those with minor injuries. This study investigated the perceived impact of skill mixed staffing, which included established NMPs in emergency departments, in the period post pandemic when patient attendances were particularly high.

**Methods:**

Semi-structured interviews were conducted and thematically analysed in six emergency departments with 35 patients and 37 staff members between September 2023- June 2024.

**Results:**

Patient participants offered views of positive impact of teams with NMPs in the context of long waiting times but were concerned that all were appropriately trained, supervised and that they were informed of their role. Staff participants also perceived a mix of positive and challenging impacts on aspects of service efficiency, safety, effectiveness and acceptability. Those participants who reported the most positive impacts on patient experience could also identify some challenging issues, for example on the workload of the senior clinical decision makers in supervising both NMPs and resident doctors. Conversely those who reported the most negative impacts, such as potential loss of training opportunities and jobs for doctors, also identified some positive impacts on the service efficiency and staff experience.

**Conclusions:**

The deployment of NMPs in ED/UTCs requires careful planning so the perceived efficiency benefits can be maximised without jeopardising quality of care, safety, and staff experience. Consideration of underlying explanatory mechanisms led to theorisation of the concept of balance as important to the optimal skill-mix within emergency department staffing. The findings suggest that the elements that require attention from clinicians and managers within the complex system of skill-mix staffing in ED/UTCs include balance between patient volume and staffing, between numbers and capacity of senior clinical decision makers and junior clinical decision makers; and between provision of service and provision of training.

**Supplementary Information:**

The online version contains supplementary material available at 10.1186/s12913-026-14832-4.

## Background

Emergency Departments (EDs) internationally have struggled to meet increased patient demand in the context of staffing shortages, particularly doctors, both before and since the global pandemic [1]. There have been multiple descriptions from different countries of the negative consequences for ED patients, staff and services [2–4]. As a policy response to global shortages and maldistribution of doctors, the World Health Organisation (WHO) has advocated the greater use of mid-level professionals or non-medical practitioners [NMPs], such as nurse practitioners (NPs) and physician assistants/associates (PAs), who are trained to undertake some of the clinical work of doctors [5]. 

### Evidence to date

A recent review of international evidence of skill-mixed ED staffing including NMPs identified that several countries have EDs employing NMPs including NPs, advanced practice paramedics and PAs [6]. A few countries have only single published reports of short-term pilot project employment of single NMPs in EDs [7–9] suggesting the employment of NMPs is not common. The review found multiple reports over time from Australia, Canada, the United States (US), and the United Kingdom (UK) where NMPs have been employed in EDs more widely to address problems in meeting increasing patient numbers and shortages of doctors [6]. However, the review reported that NMPs were not universally employed in EDs in these countries, even in the US which has a 50-year history of employing NPs and PAs in EDs [10]. There was published opinion and study evidence from the US and Australia that the employment of NMPs was contentious and not all emergency medicine doctors, nurses and managers supported the employment of NMPs. In these publications questions were raised about the lack of clarity of the role; requirements for medical supervision, and lack of evidence on quality and cost [11–14]. A recent study in England demonstrated a disjuncture between national policy positively supporting NMPs in EDs and Urgent Treatment Centres (UTCs, which provide urgent medical help when it’s not an emergency), and the low levels of implementation in individual hospitals [15]. 

### The evidence gap

Much of the evidence on NMP clinical effectiveness has been observational from a single site comparison of NMPs substituting for doctors for certain types of consultations, without consideration for health economics [6] and without broader consideration of the NMPs as part of a skill mixed staffing team in a consultant-led 24-hour, 365-day ED service. The international evidence on the impact of including NMPs (mainly NMPs from the nursing profession) in ED/UTC staffing reports neutral or positive evidence, primarily reporting on ED processes rather than patient or staff outcomes [6]. To address this evidence gap, we report here our study which investigated the question: what are clinical staff and patients’ perceptions of the impact for patients and staff of including NMPs in the overall skill mix of ED/UTC staffing in England. The study was part of a larger mixed methods, multi-phase study (known as the parent study in this paper), which included analysis of national quantitative data of skill mixed staffing configurations and service and patient outcomes [16]. 

## Methods

### Aim

The aim of this study was to explore perceptions among ED/UTC patients and clinical staff of the impact of including NMPs in the skill mix of the staffing.

The study drew on the interpretivist tradition in qualitative research which recognises that there are multiple perspectives on the same social phenomena e.g. events and relationships, and that these multiple perceptions occur within specific socio-cultural and historical contexts [17]. The study therefore sought to explore the perspectives of diverse groups working in and attending the ED/UTCs.

Impact is a broad term, but this study employed it in terms dimensions of quality in health care for patient populations. These dimensions address structure, process and outcomes through concepts such as access, clinical effectiveness (including patient safety), acceptability, equity (fairness in provision), efficiency and cost [18]. Impact can therefore be viewed in positive and negative terms as well as a mixture of both.

### Health care setting for NMPs in England at the time of the study

The English National Health Service (NHS) is tax funded, and free at the point of care. The NHS is directed by government policy in its overall design and delivery of services as well as in the provision of its workforce. English NHS policy, developed with the Royal College of Emergency Medicine (RCEM) since 2017, has supported the development of multi-professional NMPs (including nurses, paramedics and physiotherapists as ACPs; and PAs) in EDs/UTCs, although RCEM withdrew support for expansion of PA roles in 2024 [19], The NMPs work variously in the different ED/UTC sections of NHS hospitals. They are expected to undertake patient histories and clinical assessments from which to develop investigation and treatment plans for agreement with the supervising consultant. ACPs are considered to be working at the equivalent level of a resident doctor in the second foundation year. ACPs who have longer years of experience and are credentialed by the RCEM (known as EM-ACPs), have competencies equivalent to specialist resident doctors at stage 3 of core and specialist training to be emergency medicine consultants [20]. Analysis of NHS England workforce data for ED/UTCs from 2017 to 2023 has shown a small but increasing number of NMPs employed [21]. Following the pandemic, there were significant increases in patient attendance at NHS EDs. Some of the highest attendance figures in the NHS were recorded in 2023 and 2024, which coincided with a period of industrial action by doctors, nurses and other health professionals [22]. In the same years, there were questions raised in Parliament, and by medical organisations, as to the safety, lack of clarity of role and impact on doctors’ training of employment of PAs in the NHS, and in December 2024 the Secretary of State announced a national review of PAs [23]. This study was undertaken within this policy and political context.

### Design and setting

The study design was within the qualitative, interpretivist tradition and used semi-structured interviews to gather data.

The study methods were informed by a patient and public involvement (PPI) group (full details of the PPI group recruitment and active involvement are published elsewhere [24]), a NMP practitioner group and a study advisory group (comprising of public representatives, clinicians, health service managers as well as academic experts in workforce research). These three groups were consulted on the study design as well as the analysis and interpretation of the findings.

The investigation was undertaken at six EDs in NHS hospital organisations, known as trusts, in England. The six EDs were dispersed geographically across the north and south of England. The EDs were purposively recruited to provide variation in setting and population by indicators of socio-economic deprivation [25], urban and rural location [26], population ethnicity [25] and in staffing composition that included proportion of NMPs (Table 1).

**Table 1:** Characteristics of study sites

Study Site	Hospital trust index of multiple deprivation [25] *	Hospital trust rural / urban classification [26] **	ED catchment area ethnicity profile [25] ***	Study applied skill-mix ratio group for density of NMPs****
1	Between 21–25	3	White 85–95%, Black < 5%, Asian < 5%	4
2	Between 10–15	6	White 75–84%, Black < 5%, Asian 6–15%	5
3	Between 16–20	6	White < 75%, Black 6–15%, Asian 6–15%	4
4	Between 16–20	2	White > 95%, Asian < 5%	6
5	Between 21–25	6	White < 75%, Black 6–15%, Asian 16–25%	5
6	Between 10–15	6	White 75–84%, Black 6–15%, Asian 6–15%	1

*Higher score = higher levels of multiple deprivation

** 1= lowest sixth for rural area, 6=highest sixth for urban area

*** Percentages given in bands to maintain anonymity

**** Proportion of NMPs in staffing as determined by previous national workforce data analysis; 1=lowest proportion of NMPs group and 6=highest proportion of NMP group

### Participants and recruitment processes

We aimed to recruit up to eight clinical staff and eight patients from each study site (total up to 96). The target sample size was guided by the concept of information power [27]. 

Patients were convenience sampled to ensure they were interviewed close to the date of their ED/UTC visit. In designated sessions, spread over three months, NHS research nurses in the six sites approached patients, on their discharge or transfer from the ED/UTC, with invitations to participate in the parent study [16]. The eligibility criteria for the NHS research nurses to approach patients were that: the person or their named advocate was aged 16 or over; clinically stable; well enough to be approached; and with capacity to consent. One part of the parent study invitation was to volunteer to be interviewed. The NHS research nurses then sent to the University research team the contact details of 192 patients who indicated a willingness to participate in an interview and consented to the sharing of their contact details for that purpose. Patients, who had consented to receive further information from the research team, were sent a more detailed interview invitation, including a study information sheet outlining the study purpose and what participation would involve, and a consent form. No payments were planned, but to help increase participant numbers a £20 voucher was offered to each participant in the final two study sites. Participants could choose to be interviewed by telephone, online, or live text chat. If a patient participant required language translation assistance from a carer or family member, efforts were to be made by the study team to enable this. The University research team sent detailed invitations by post or email to 188 patients (six had not provided legible or complete contact details). 41 patients responded to invitations and provided a phone number or email for the research team to contact them to arrange an interview time. Reminder invitations were sent after two weeks to non-responders. It was not possible to collect reasons from the six patients for their subsequent non-response. All patients who volunteered and responded to arrange an appointment were interviewed.

Recruitment of staff participants was aimed at obtaining doctors, nurses and NMPs with a broad spread from across junior and senior clinical and managerial/administrative roles. A senior clinical manager in each study site sent all staff who met the inclusion criteria an email invitation to participate in an interview. Those staff who were interested in participation were asked to contact the study research team directly, to ensure the clinical managers had no role in consent and to avoid any pressure on staff to participate. The eligibility criteria specified consenting junior to senior clinical staff, and managerial/administrative staff working in the ED/UTC. Staff were offered a £35 voucher for undertaking an interview in their own personal time. The University research team sent detailed invitations, with information sheets and consent forms by email to 59 staff. Of these 59 staff, 22 did not reply or respond to a reminder email. It was not possible to establish the reasons for this. All staff members who volunteered and responded to arrange an appointment were interviewed.

Recruitment and interviews were undertaken over a period of nine months (September 2023- June 2024). Consent was obtained in writing and/or via digital recording prior to the interview, as preferred by the participant.

### Data collection

The semi-structured interviews consisted of open-ended questions with supplementary prompts to enable key issues to be explored without being prescriptive [28]. Topic guides were developed based on the study aims and informed by input from the PPI, NMPs and advisory groups [see Additional file 1]. The patient topic guide included questions about the type of care received and from whom, how staff team members worked with each other (inter-staff communication, independently or with supervision), staff skill-mix including NMP roles, communication with the patient, being treated without harm, and satisfaction with treatment. Staff were asked about their experiences and perspectives on NMPs in the skill-mix team, including their interdependence with other staff, what worked well and less well, and whether there were any perceived benefits and/or problems.

Four female members of the research team conducted the interviews: three with prior experience of research on NMPs and one a clinician. All interviews were audio-recorded using digital voice recorders and professionally transcribed verbatim, with transcripts proof-read against recordings. The transcripts were anonymised, and the recordings deleted.

### Data analysis

A framework approach was used to code and categorise the interview data [29]. An initial deductive coding framework was developed by four study researchers as well as a patient peer researcher, based on process and outcome impacts identified in the study scoping and systematic review [6]. A sample of patient and staff transcripts were read, re-read and coded inductively by two or more researchers to add to the deductive coding framework to create a coding framework for patient interviews and a separate one for staff interviews. Data were broken down using line-by-line coding and the codes clustered manually to identify preliminary inductive codes and categories. These coding frameworks were discussed within the research team, disagreements resolved, and the codes iterated. Codes included in the two frameworks were refined and elaborated collectively with data collected from further interviews, some combined and others removed. Every transcript’s coding was reviewed for consistency by one researcher. NVivo v.14 (QRS International) supported coding. A patient peer researcher was involved in coding, bringing lived experience to the process and challenging some interpretations which led to reconsideration of how data were categorised. A comparative analysis was undertaken between the data sets from patients, doctors, nurses and NMPs. A further stage of synthesis was undertaken in discussion with all research team members to triangulate and interpret findings, looking for patterns, differences, and explanations across the data sets.

### Reflexivity

The research team was mixed in their previous experience of undertaking research in relation to NMPs in the health care workforce. Some members had no previous experience of such studies while other members had conducted several studies. This mix of prior experience was a strength in ensuring a range of analytical perspectives and reducing any bias in approach, analysis and interpretation. The rigor the study was further enhanced by the involvement of the PPI, NMP and advisory groups. In meetings with the research team, the PPI, NMP and advisory group members were able to further interrogate and test the analysis and the interpretation of findings. Their viewpoints were included in the final synthesis reported in this paper.

The paper conforms to the standards for reporting qualitative research [30].

## Results

Seventy-two interviews were undertaken: 35 with patients and 37 with staff from across the study sites (Table 2). Most participants chose to be interviewed by phone (89% patient participants and 62% staff participants), the remainder were interviewed online using Teams™ or Zoom™ dependent on participant choice. None of the participants selected to be interviewed by live text chat. Interviews lasted between 20 and 48 min, with the median 34 for patient interviews and 36 min for staff interviews.

**Table 2:** Participants by study site

Study site	Patient participants (*n* = 35)	Staff participants (*n* = 37)
1	3	5
2	8	6
3	6	3
4	4	6
5	9	9
6	5	8

### Participant characteristics

Patient participants were aged between 23 and 89 years with the majority female (60%) and of white British ethnicity (61%, information was not available for five participants). There was diversity in the ethnic backgrounds of the remainder including Black Caribbean, British Bengali, British Indian, and British Pakistani. One participant represented a patient as their carer. The patient participants’ route into the ED/UTC included six walk-ins or self-presentations, six via ambulance, six via general practitioner (GP, known as family physician in some countries} referral, three via NHS 111 (an NHS telephone helpline), and two from other parts of the same hospital. Eight patient participants mentioned being attended by an NMP, and thirteen mentioned being attended by staff that were not NMPs. Twelve of the 35 patient participants reported working for, or previously working for, the NHS.

Staff participants included 14 medical staff, 9 nursing staff, and 12 NMPs. They variously worked in ED/UTCs that employed: (a) only ACPs with mixed professional backgrounds, (b) only ACPs with nursing backgrounds, and (c) ACPs and PAs, as well as ENPs/EPs staffing the UTC for most sites. The 14 medical staff participants were diverse on a range of characteristics: (a) length of time working in EDs/UTCs (for over 25 years to a few months), (b) medical seniority (from senior consultant with responsibilities for medical education and service management, to GPs, to resident doctors in the second year of postgraduate foundation programme) and (c) experience of working in different EDs/UTCs (from multiple during their postgraduate medical training, including in other countries to a single ED/UTC experience). The nursing staff participants ranged in experience and responsibility from NHS salary band 3 (a health care assistant) to NHS salary band 8 (a senior sister). The length of time they had experience of working in EDs/UTCs varied from three to 25 years. All medical and nursing staff participants had experience of working with NMPs, mainly nurses in advanced clinical practice roles.

Of the NMP participants: six were ACPs and registered nurses (2 were also nurse consultants), three were PAs and three were ENPs. Their experience of working in EDs/UTCs as NMPs ranged from 1 to 15 years. The NMPs variously described working in different sections of the ED, some of them in paediatric sections as well as adults (acute assessment units, majors, resuscitation) and the UTCs.

We now present the findings reporting on the perceived impacts first on service and patient experience and then on staff. We present this comparatively across the types of participant groups. Integrating the findings in this way enables insight into where perceptions intersect, identifying interconnections and differences between the groups. Verbatim quotes are provided as exemplars to support the points made. Further verbatim quotes are provided in Additional file 2. It should be noted that participants tended not to use the term non-medical practitioners but used more specific terms such as ACP, nurse practitioners and PAs. We conclude the results by reporting on the views of staff as to the ideal skill-mixed staffing team in ED/UTCs.

### Perceived mixture of impacts

All patient and staff participants reported a mix of positive and more negative, or challenging impacts. Those patient participants with experience of working in the NHS reported predominantly positive impacts. Those staff who reported the most positive impacts could also identify some challenging issues and conversely those reporting the most negative impacts reported some positive impacts.

*I’m very positive about it *[having NMPs in the staffing]*…. I think they clinically make good sound decisions*,* and I think they are definitely positive within the workforce…. I think the limitations are the amount of time it takes to supervise them and the amount of time it takes …to get through the credentialling process.* Emergency medicine consultant, 2079

*I thought she explained very well. As I say*,* just before I was discharged*,* the nurse practitioner explained to me what she believed it was and why it had happened*,* and then she went to get the okay from the lead consultant……In an ideal world*,* I would have preferred to have seen the senior consultant*,* just purely because she said*,* because of how I presented and my age*, etc.,* she needed to get the sign-off from the senior consultant*. Patient 2380

*The ones* [ACPs] *that are competent*,* the ones that are working well*,* I feel that they’re doing a good job with it. …. So if you have a look at all the medical staff*,*….*,* and even the first very inexperienced of them* [foundation year doctors] *on their first day*,* will do a better job than your average ACP*,* okay.* Resident doctor -senior house officer, 5416

One possible explanatory theory for such mixed views may lay in the variation and lack of uniformity within the NMPs’ staff group, we explore this point more fully in the discussion. We turn first to look at specific areas of perceived views of impact.

### The service and patients’ experience: perceived impacts

**Fig. 1:**
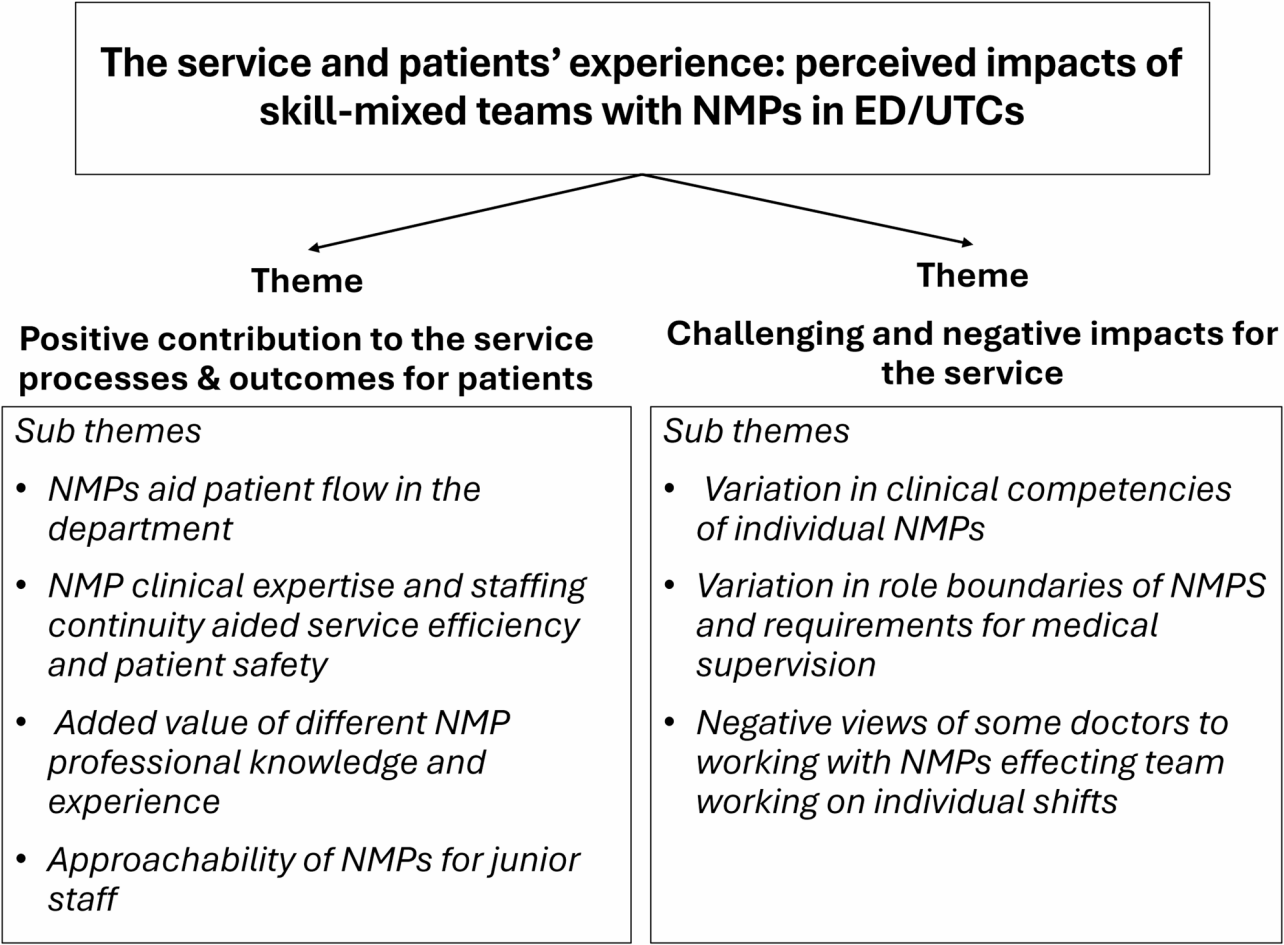
Themes and sub themes of perceived mixed impact of skill mixed teams with NMPs in ED/UTCs on the service and patients experience

The perceived impacts on the service and patients’ experience can be grouped into two themes, that of perceived positive and perceived challenging or negative impacts. Each theme has sub themes which are now discussed in turn. These are presented schematically in Fig. 1. Additional file 2 provides more quotations.

#### NMPs perceived to aid patient flow in ED/UTCs

All four participant groups (patients, doctors, nurses and NMPs) discussed the positive contribution NMPs made to the service in dealing with the high and increasing volume of patients in the ED/UTCs. The positive impact most frequently commented on was the concomitant reduction in wait times for patients and consequent improved flow of patients through the department.

*But when you’re sometimes waiting for five hours*,* it is a very long time. So*,* if there are different team members that can take the pressure off and help reduce that*,* obviously it is generally great for people that are waiting*,* even if it just helps the time by half-an-hour.* Patient, 2226

*Yes*,* I think it’s a positive impact. I can see for me*,* like the more hands can help in the department*,* the better you’re going to be. If tomorrow we just stop having ANPs in the department - it is going to be a negative impact for sure.* Resident doctor-junior clinical fellow, 2210

Some doctors and nurses also discussed the positive impact NMPs had on the medical workload, which also helped with patient flow,

*When we work in the majors*,* they* [NMPs] *are working with us*,* we are colleagues*,* so they give us a great hand*,* yes*,* many patients we are seeing*,* and many they are having.* Resident doctor-clinical fellow in emergency medicine, 3484

Patients too speculated that this was part of the NMPs’ impact:

*Well*,* I think they take a lot of the doctors’ work off them so that they can focus on other things.* Patient, 4952

Some of the NMPs observed that they were able to be flexible in their work to cover the work of nurses in order to help with patient flow.

*Doing bloods and putting in catheters*,* doing a set of obs* [observations], *taking cannulas out if the patient is going home*,* they are not nursing-specific jobs*,* and I frequently do those for pretty much all of my patients because the nurses don’t have the time to do it and I’m very happy to do it.* NMP (PA),3729

The period in which this research was undertaken was one of intense, highly publicised, pressures on EDs throughout the UK, with some of the highest levels of patient attendance and wait times ever recorded by the NHS [22]. It is therefore perhaps unsurprising that participants commented on the NMPs’ contribution to ensuring timely attendance to patients and the patients’ progress to discharge from the ED.

#### NMP clinical expertise and staffing continuity perceived to aid service efficiency and patient safety

Doctors, nurses, NMPs, and some patients who were also NHS staff, identified that because the NMPs were employed long-term in an ED/UTC, they provided continuity in staffing with detailed knowledge of that hospital’s protocols, care pathways, processes and staff. The NMPs were therefore perceived to contribute positively to efficiency and safety in delivering patient care, again assisting with patient flow.

*Foundation doctors rotate every three to six months*,* so you’re having to teach them all again where everything is…and then the registrars sometimes rotate yearly…. Whereas they’re* [NMPs] *are permanently in the department… and they know all the pathways and protocols and how to do referrals.* Nurse, 4970

Some doctors, nurses and NMPs commented that this positive contribution was underpinned by clearly defined departmental work boundaries and levels of supervision for NMPs. A number of NMPs commented that the NMPs new to the department were more closely supervised by senior doctors than those NMPs who had worked there for longer periods. NMP work boundaries and sufficient supervision were issues raised in the challenges by some doctors and are discussed further in that section.

It should be noted that there was also positive endorsement by patients and staff of the contribution ENPs made in the UTC part of the ED in attending to patients with minor injuries and illnesses.

Some doctors provided testimony to the high quality of the clinical expertise of the NMPS that they worked with which benefited the patients. The doctors attributed some of this clinical expertise to the extensive years of working in EDs.

*They’re* [NMPs] *incredibly competent and actually*,* I think they are more competent than some of the senior house officers we get*,* and indeed some of the registrars.* Resident doctor *-e*mergency medicine speciality doctor, 4688

The years of experience was also linked to the view that NMPs provided continuity in staffing which in turn contributed to patient care outcomes and patient safety.

#### Perceived added value of different NMP professional knowledge and experience added value in clinical assessment and planning

Some doctors, nurses and NMPs stated, and some patients speculated, that the NMPs brought added value to the assessment of patients and care planning. The added value was attributed to knowledge from the NMPs’ previous professional role, such as physiotherapist, or to their expertise with certain patient populations such as the frail elderly and those with mental health problems.

*I think it’s good to get different opinions from - and especially people that are actually seeing the patient. When you do actually get to see a doctor*,* it can be very fleeting….Whereas I think if there’s more time for a conversation to be had from the people that are directly looking after a patient*,* then I think that’s a good way forward.* Patient,6883

*So it’s like helpful for the overall management of the patient*,* like they’re* [NMPs] *involved in the direct patient care*,* especially in frailty patients. They can assess. They are more experienced in handling the frailty*,* elderly patients. Also in mental health issues they are more experienced.*, Resident doctor-speciality trainee level 3, 1339I

#### Approachability of NMPs for junior staff

A further aspect of added value commented on by some nurses, a doctor and NMPs themselves, was that the NMPs were perceived by many nurses as a more approachable clinician than some of the doctors. This perceived approachability was thought to improve communication and teamwork for the patients’ benefit.

*They* [nurses] *come to us for everything. Constantly*,* I’ve got somebody going*,* ‘Oh*,* the doctor over there is too busy. Can you prescribe this for the patient? Can you check this ECG? …. I probably say*,* ‘Yes*,*’ a bit more often than our doctor colleagues*. NMP (nurse), 1434

*what I’ve found*,* especially in the UK*,* is there is a*,* a bit of a barrier between the nurses and the doctors…. a bit of hostility….But I think having that advanced practitioner working with us as a clinician helps break down those barriers and work as a team together* Resident doctor -registrar, 1365

Some nurses commented not just on approachability but on NMP willingness to listen to nurses’ concerns about patients.

*Specially from the nursing background*,* the ACPs*,* I can go up and go*,* I’m just not happy about this patient and they’ll come and go right ‘we’ll come and see him and do a bit more investigation into why’. Like they understand what I mean.* Nurse, 4970

Health care organisations are complex and hierarchically organised systems. The position of individuals in the organisation and in health care teams are hierarchically structured, linked to status and power, both within and between health professional groups [31]. These dynamics are known to influence aspects of teamwork and patient safety and NMPs have been noted before to provide a bridge between medicine and nursing [32]. 

#### Variation in clinical competencies of individual NMPs

Many of the medical participants commented on the variation in the knowledge and skills of different NMPs and the challenges this created for them. Some doctors affirmed that where ACPs were RCEM credentialled, they understood the ACP’s abilities in emergency medicine and viewed them as very competent, “*They’re* [ACPs with RCEM EM-ACP credential] *incredibly competent*,* …. I think they’re exceptional clinicians*”. Emergency medicine speciality doctor 4688.

Many doctors and all the NMPs recounted that their department policies required close supervision for newly qualified, or new to the department, NMPs. However, many of the doctors were working with NMPs who had variable training and clinical competencies in emergency medicine, and this had implications for levels of delegation, supervision and oversight by the doctors.

*So indeed*,* you will be seeing their* [NMPs] *clinical skills on and off*,* but we* [doctors] *are not aware which level they are. Like we have F1*,* F2*,* SHO*,* and registrar… They* [NMPs] *are not defined according to experience and their qualifications …but not knowing their stage*,* it’s a problem to give responsibilities*,* like when we’re* [doctors] *handing over the patient.* Resident doctor speciality trainee level 3, 1339

As noted earlier in the paper NMPs came from multiple professional backgrounds and through varied routes to their employment in EDs. This sub-theme of variation in clinical competencies linked to the next sub-theme of variability in role boundaries.

#### Variation in role boundaries of NMPS and requirements for medical supervision

Many doctors and some NMPs commented on the challenges of NMP role boundaries. One dimension of this was problems with the legal limits to roles, for example, PAs not having the legal authority to prescribe medication or order ionising radiation and ACPs not having gained the qualifications for independent medication prescribing. Doctors, nurses, NMPs and patients who were NHS staff commented on the inefficiencies caused by some NMPs not having legal authority to prescribe medications.

*Let’s say we’re very short-staffed and we’ve got one doctor and then one PA. Including the nurses’ requests*,* whatever they need a doctor to do*,* the PAs would also have to wait for the doctor to be able to prescribe*,* or to request an x-ray. All of that happens while a doctor’s also looking after their own patients. It’s just the delay.* Senior nurse, 6876

Other examples given were of potential delays in patient care that were time critical such as the treatment of sepsis.

A few of the doctors were also concerned about their own medic-legal position and responsibilities in such situations as authorising medications or ionising radiation for patients presented to them by NMPs.

*I often feel quite uneasy about prescribing on behalf of PAs*,* and requesting any radiation on behalf of them*,* because I know that it’s my GMC number against that request for a patient that I don’t know.* Resident doctor -foundation year, 6862

Some of the doctors reported that, on occasion, they repeated the NMP’s assessment of the patient, in effect double handling those patients.

Interrelatedly, a few doctors expressed concerns about some NMPs working beyond their competencies on occasion, which was also noted by an experienced NMP.

*I’ve had some experiences where non-medical practitioners didn’t know the limitations of their competencies and have sometimes overstepped the boundaries*,* where they would just make independent judgements. And sometimes that can - that has been detrimental to patients.* Resident doctor -registrar, 1365

The senior consultant participants commented that there was variation also in the competencies and confidence among junior resident doctors.

Some patients speculated that they would feel uncomfortable if NMPs strayed into working in ED/UTC areas for which they were not qualified.

*I would be uncomfortable if they* [NMPs] *strayed into the areas of medicine which has currently been the province of doctors who are qualified to deal with issues.* Patient, 5327

It should also be noted that some doctors and nurses suggested that at times the sheer volume of patients, or the limited availability of doctors such as on night shifts, resulted in NMPs widening their scope of work to help meet the demand in a timely manner.

Healthcare professions have never had static role boundaries; rather the professions’ role boundaries are dynamic in nature [33]. Dynamic role boundaries have the potential to challenge the roles and boundaries of other groups and in this give rise to conflict and protectionism [32, 33]. The next sub-theme provides evidence of some of these tensions.

#### Negative views of some doctors to working with NMPs effecting team working on individual shifts

Some of the doctors, nurses and NMPs recounted that there were individual doctors who held openly negative views about NMPs. These negative views were reported to impact on team working processes. The NMPs and some doctors described the NMPs gravitating towards working with those senior doctors who viewed their role more positively and avoiding those who didn’t. Doctors, nurses and NMPs recounted increased hostility from doctors towards PAs during the period of data collection. The PA NMPs described passive aggressive behaviours by doctors towards them. A letter from the British Medical Association (the doctors’ trade union), to its members, which advised doctors not to sign PA requested medicine prescriptions or requests for ionising radiation, was reported as increasing problems in patient care in some departments.

*Yesterday a junior doctor refused to sign a prescription for paracetamol for a PA’s patient. The consultant saw and came over and signed it. It was very difficult for everyone. The PAs are scared*. Researcher’s contemporaneous notes of interview with Senior Nurse, 6870

While discussion on disputes about role boundaries between professional groups tends to be at level of professional organisations and national agencies [31–33], participants in this study describe the impact at the health care team and service delivery level. In part they described the impact of national role disputes on individual staff members. We turn now to report more specifically on the perceived impacts for staff of skill mixed teams with NMPs.

### The staff experience: perceived mixed impact of skill mixed teams with NMPs in ED/UTC

**Fig. 2:**
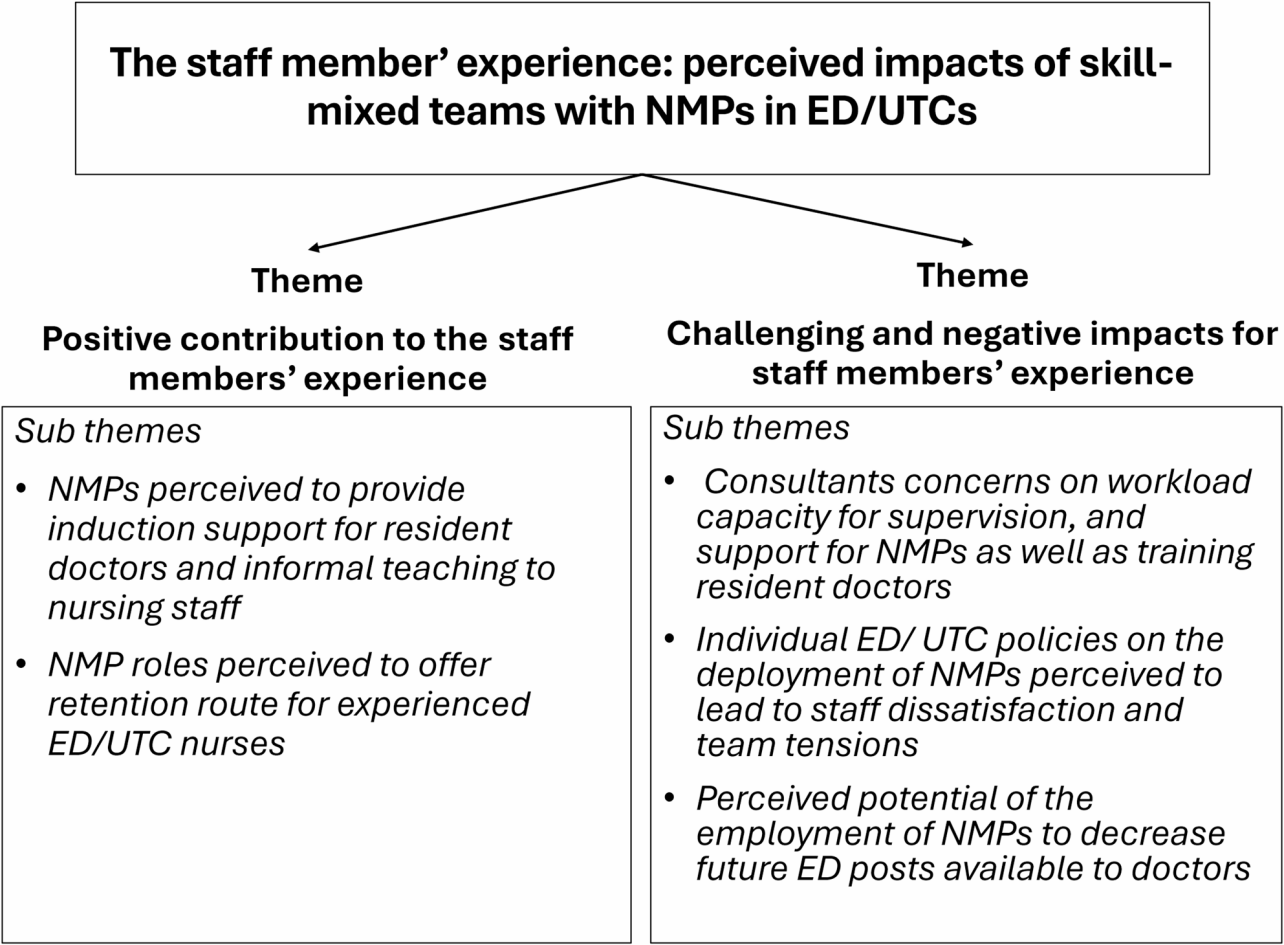
Themes of staff experience perceived mixed impact of skill mixed teams with NMPs in ED/UTC

The perceived impacts on the staff members’ experience can be grouped into two themes, that of perceived positive and perceived challenging or negative impacts. Each theme has sub themes which are now discussed in turn. These are presented schematically in Fig. 2. Additional file 2 provides more quotations.

#### NMPs perceived to provide induction support for resident doctors and informal teaching to nursing staff

Many staff participants identified the support given by NMPs to the induction of new resident doctors as a positive for those staff members experience. This support was described as help in induction to both resident doctors new to the department and those doctors who were also new to emergency medicine.

*Some other positive things about PAs*,* because they’ve been in the emergency department*,* and as a foundation doctor*,* we only spend four months in each rotation*,* so we have to learn very quickly*,* and because they’ve* [PAs] *often been there for a number of years*,* they know how everything works and they can be a good resource when you have any questions but don’t perhaps want to go to the consultant.* Resident doctor -foundation year,

One doctor also speculated that overall, the support of NMPs to the medical team might help prevent the ‘burnout’ of some of the doctors.

Some of the nurses commented on the willingness of the NMPs to help expand their knowledge and this was seen positively.

*The PAs*,* I’ve found*,* are really good at teaching. They really*,* really enjoy saying*,* ‘Oh*,* you’ve got a question about this medication; well*,* I’ll tell you exactly the pathophysiology behind it*,* and how it works*,*’ and all of that.* Junior nurse, 6872

EDs in the UK are typically staffed by a small group of permanently employed doctors (some of whom may be part-time or sessional only), and a larger group of doctors in training who are placed there for relatively short periods of time. The resident doctors at the generalist foundation years of their training work in the ED for a few months only, as do doctors training for other specialities. Doctors at different stages of training to be emergency medicine consultants will also rotate between different hospitals and EDs. There is therefore frequent change in the medical team many of whom will not have worked at that hospital before [34]. The induction and support needs of these doctors on placement rotations are therefore considerable, and it is unsurprising that the NMPs as permanent ED staff were seen as an asset to the staff and service in this context.

#### NMP roles perceived to offer retention route for experienced ED/UTC nurses

Some doctors and nurses observed that having NMP roles offered a clinical career route that retained some experienced nurses in emergency departments to the benefit of the staff and the services.

[NMP roles] *helps with the retention of the nursing staff and also gives them an opportunity to do - to become more clinical*,* as compared to being limited to their role*,* because in terms of progression in nursing you become more and more managerial rather than clinical.* Resident doctor- registrar, 1365

It should be noted that a few of the nurse participants did not view an NMP role as attractive for a variety of reasons. These reasons included concern about the level of clinical responsibility, the cost of education and a perceived lack of career progression for NMPs.

#### Consultants’ concerns on workload capacity for supervision, and support for NMPs as well as resident doctors

Some consultants and GPs voiced concerns about their workload and their capacity to supervise NMPs and the resource consequences for the service. The GPs additionally raised questions of responsibilities in terms of their employment with the organisation. Emergency medicine consultants were also concerned about workload capacity to educate and assess trainee ACPs at the same time as resident doctors.

*it’s no good throwing in two more ANPs into ED and not really job plan your consultant workforce for the additional commitment to supervision*,* training and so forth…… an assessment with an ANP trainee might take double the time it would with a medical trainee.* Senior emergency medicine consultant, 1740

The process of credentialling EM-ACPs through RCEM was viewed as particularly onerous on consultant resources.

All NHS services function as a training facility for future generations of all health professionals at the same time as providing a service to patients. There has been considerable attention paid within the NHS policy arena for staff to have good quality clinical, workplace and educational supervision although these policies rarely state the senior staff time resource required for these types of supervision or when the supervision crosses professional groups [35]. 

#### Individual ED/ UTC policies on the deployment of NMPs perceived to lead to staff dissatisfaction and team tensions

The six study sites had different deployment patterns for their NMPs. Consequently, some doctors stated that NMPs did not work night shifts which was a problem for the service because of the difficulties in covering medical rotas. “*they* [NMPS] *are I think not doing night shift…. Sometimes it is a problem covering nights*,* like ED doctors and medical staff”*, Resident doctor, 1339. It should be noted that the NMPs interviewed reported that they and their colleagues were working weekend and night shifts. In some sites NMPs were mainly deployed to sections such as the first assessment area or similarly named section. Deployment in this section was considered by NMPs and doctors to involve high intensity, but repetitive, work. Some participants considered that deployment only to this area was likely to contribute to NMPs leaving their jobs and thus having cost consequences for the service in replacing them. Other NMPs and senior doctors described distribution of NMPs to a variety of sections of the ED during shifts and for specific parts of rotas. While these doctors and NMPs considered placement in a variety of sections as positive for the service, other doctors pointed to this as a source of tension in the team and service, particularly with reference to perceived restriction to their training opportunities.

Many of the senior doctors emphasised the importance of ensuring resident doctors were able to undertake the required training and assessments in the relevant sections of the ED. However, there were resident doctors who reported consultants rostering NMPs, rather than resident doctors, to staff certain areas, either because they thought the consultants were used to working with the individual NMPs, or because the NMPs too required training. It was evident that this issue was a source of tension in both individual shifts and the wider service.

*When I first started at this Trust*,* I was struggling to get time in resus [the resuscitation area of the ED] because the ACPs needed time in resus to fulfil their training requirements… So*,* I’ve lost my training opportunities……. Yes*,* I feel like I’m less important than an ACP* Resident doctor-speciality trainee level 1–2,5270

EDs internationally are recognised as stressful work environments resulting in problems with staff retention [2–4]. One strategy in many EDs is to ensure variety in the sections that staff work in, in order to reduce stress and increase job satisfaction. The challenge raised for senior staff is how to address these issues for those permanent staff providing a service and those staff who are providing a service but also expecting to fulfil their training requirements.

An additional issue raised by several doctors, and some of the senior nurses, was the exclusion of resident doctors from the minors’ section in some UTCs, which were solely staffed by ENPs. The consequence was reported to be resident doctors who had little or no experience in undertaking the clinical procedures commonly undertaken in UTCs such suturing minor injuries or plastering fractures. Resident doctors without suturing or plastering skills were reported to create problems for patient care when the UTCs were closed.

Innovation in the health care often has unintended consequences. These participants provide an example of such in workforce when skills and knowledge of specific procedures are shifted from one group to another group of staff without full cognisance of longer-term consequences.

#### Perceived potential of the employment of NMPs to decrease future ED posts available to doctors

A few of the doctors considered that the employment of NMPs was likely to negatively impact the job opportunities for doctors in a health care system facing significant financial challenges.

*I think the impact is in the years to come*,* if there is more nurse practitioners going into the field*,* there are going to be less doctors*. *I don’t see the benefit*,* but I am just looking through my lens. Maybe they cost less for the Trust…. The ACPs are useful*,* but they’re not doing anything extra or anything that the junior doctor wouldn’t be able to do. Resident doctor -junior clinical fellow*,* 5416*

The commentary here is one of future competition between the professional groups for jobs in emergency departments. The participants here reflect the wider political environment of the time when the medical professional bodies and unions were actively campaigning not only for improved pay and conditions for doctors but for limitations to NMP type roles [23]. This is enlarged upon in the discussion section.

### Views on the future skill-mix of staff required in ED/UTCs

Many staff and patient participants responded to questions about views on the future skill mix of staff required in EDs/UTCs in the context of the major challenges facing the service at the time of the interviews.

*All you’ve got to do is turn on the TV and you see NHS waiting lists and the fact the doctors were on strike*,* the nurses were on strike. …yes*,* the NHS gives the impression it’s on its knees and things need to change desperately.* Patient, 2380

Patient participants could see the value of having a variety of different skill levels in the team but were also concerned that newer types of staff were properly competent, supervised, and the public informed about them. However, patient participants offered less comment on this topic, with their responses focussing more on individual experiences than on overall skill-mix.

*where a lot of people who don’t need to be sat in front of a highly skilled doctor/consultant could go and have intermediate care. You know*,* they get to triage says right*,* go over and see that nurse practitioner rather than stay in A&E which should be accident and emergency. It kind of might work very well I would have thought.* Patient, 5003

*The job title didn’t mean anything to me. So*,* I’m slightly concerned about that… It’s providing the people who are using the service with the information about what the level of expertise staff have*, Patient, 1653

Many of the staff participants considered the ideal staff skill mix to involve an overall increased number of staff; sometimes a specific groups such as nurses. Other participants reflected on the question more broadly. These staff suggested other non-health professionals such as social workers and social prescribers, who they considered would help provide for the range of problems that patients attended with.

Many of the doctors and a few of the senior nurses reported that the ideal skill mix should have increased numbers of consultants as senior decision makers, to aid both the service, supervision and the training of junior staff. Those doctors who had worked in other countries used their experience to contrast to their current organisation, as in this example.

*Okay*,* so the ideal mix for me*,* lots more consultants. …Say*,* as an example*,* a department of my size - or where I work - and the number of patients we see*,* if it’s in Australia*,* we’d have 35 consultants. What I have is ten consultants.* Senior Emergency Medicine Consultant 1740

One resident doctor suggested a ratio of at least ten doctors to two ACPs because of supervision requirements and a couple of the doctors considered a 1 to 1 ratio of doctors to all other types of clinical staff as their ideal. One NMP described writing a business case for increased numbers of NMPs in the context of high levels of employment of locum doctors. Following a cost cutting imperative in the organisation, all plans for new staffing were shelved including this business case.

Staff participants who responded to this question with a view of a skill mixed team which included NMPs, tended to discuss it in terms of a balance of, and between, a number of considerations. These considerations included: the volume of patients attending and their acuity; the ratio of senior decision makers to junior staff; the need to have staffing for a 24-hour 365 day-a-year service, and to have manageable numbers of students and staff in training posts to prevent friction between groups. This concept of ‘balance’ resonates with theories about health care as complex adaptive systems i.e. systems that consist of elements that interact dynamically in a non-linear manner with feedback and operate in conditions of multiple temporary equilibria [36, 37]. We consider these concepts further in the discussion section.

## Discussion

This study investigated the perceived impact of NMPs in the skill-mixed staffing of EDs/UTCs in England at a time of: soaring public demand; industrial action by health professional staff; and public questioning of some NMP roles, particularly PAs, in the NHS. In this turbulent context, we found that all staff participants offered views of a mix of positive and challenging impacts on aspects of service efficiency, safety, effectiveness and acceptability. Those participants who reported the most positive impacts on patient experience could also identify some challenging issues, for example, increases on the workload of the senior clinical decision makers. Conversely, those who reported the most negative impacts such as potentially on less job opportunities for the medical profession also reported some positive impacts on the service efficiency and staff experience. Although single site studies at early points of the introduction of nurse practitioners in EDs in Australia and in Scotland have reported similarly mixed views [38, 39], this study is the first to report such mixed views of the impact of established NMP posts in multiple sites. One possible explanatory theory for such mixed views may lay in the variation and lack of uniformity within the NMPs staff group. NMPs working in ED/UTCs come from different professional backgrounds (nurses, paramedics, physiotherapists and PAs) with different types of training and work experience in ED/UTCs. Further, in the UK advanced practice is not a regulated or standardised qualification for nurses, paramedics or physiotherapists [40, 41]. 

Patient participants offered views of positive impact of NMPs in the skill mixed team in the context of long waiting times but were concerned that all who attended them were appropriately trained and supervised and that they were informed of their role. These findings concur with other studies from the UK investigating patient views regarding PAs working in EDs, although we could not find any studies of ACPs [42, 43]. Our finding that some questions about optimal skill-mix were more problematic for our patient participants (even if they coincidentally were NHS employees) to discuss their views on skill-mixed teams was perhaps unsurprising as it this would be peripheral to participants lived experiences of ED/UTC attendance.

The positive impacts perceived by staff on patient experience, on the service and for staff members included: managing the volume of patients; bringing different clinical expertise to patient processes; induction and support of resident doctors on short term rotations; aiding communication between team members; and retaining experienced ED nurses. Each of those types of impact can be viewed as positive process contributions to the efficiency, safety and efficacy of the ED/UTC services for patients and staff. As a qualitative study our findings can only be generalised at a theoretical level for explanations to further test [44]. We have undertaken this by identifying potential mechanisms, i.e. the underlying entity generating the process outcome [45]. The potential mechanisms we have identified included: appropriate numbers of clinical decision makers; diversity in the knowledge and experience of the clinical decision makers; and stability in the clinical decision maker workforce. These possible explanatory mechanisms and hypothesised processes require further investigation through quantitative study in multiple contexts. The parent study [16] within which this study is nested is currently undertaking such analysis.

The perceived challenging and negative impacts at the service level were considered by participants to be: the variation in the clinical competencies of the NMPs; the variation in NMP role boundaries; the lack of availability of the required consultant time in supervision and training; and the negative views of some doctors towards working with NMPs and working with PAs in particular. Some, but not all, these points such as hostility to working with NMPs in EDs, have been identified in other studies from the US and Australia. [13–14] Each of these impacts could potentially have a negative impact on the team processes for efficiency, safety and efficacy of the ED/UTC services for patients and staff. We identified potential explanatory mechanisms [45] for these perceived negative impacts including: unbalanced numbers of senior clinical decision makers to juniors; variability in standards of education and regulation of NMPs; and the defence of the medical profession’s jurisdiction, which we discuss below [31]. The first theorised mechanisms require further empirical investigation through quantitative study in multiple settings, which we are undertaking as part of the parent study [16]).

A few participant doctors viewed the employment of NMPs in the ED/UTC as having both a negative impact on the training of doctors and the potential to diminish the employment market for doctors. The view that the presence of NMPs have a perceived negative impact on doctors’ training in emergency medicine has been reported before in the US [46]. In the UK impact on doctors training was an issue raised in the government commissioned review of the role of the PA and Anaesthesia Associates, although no evidence was reported to support this [47]. At the time of the research, the British Medical Association (a professional union) issued statements that doctors were unable to obtain jobs [48]. Abbott offered a view of a system of health professionals in which each seeks to enlarge and defend their own occupation’s areas of jurisdiction which is in turn linked to its status, rewards and power [31]. The defence of the medical professions’ against other occupational groups seeking to undertake aspects of doctors’ work is not a new phenomenon [33]. Opinion articles from the US reported defensive actions on the part of the medical profession within the ED setting [11, 12]. While the viewpoints reported here can be interpreted as a defence of a profession’s boundaries as in clinical work the views can also be read as a defence of jobs for doctors [31]. It should be noted that the doctors in this study were divided in their views about NMPs, this has previously been reported in the UK setting in a study of PAs in primary care [32]. Longitudinal study is required to investigate the growth or otherwise of NMPs employed in ED settings.

In identifying the optimal skill mix in ED/UTC teams, the concept of ‘a balance of considerations’ was identified from participants’ views. This concept resonates with theories about health care as complex adaptive systems i.e. systems that consist of elements that interact dynamically in a non-linear manner with feedback, and operate in conditions of multiple temporary equilibria [35, 36]. In this study the concept of balance or equilibrium was identified in a number of dimensions: the balance between ensuring timely patient flow and ensuring appropriately trained and supervised staff attend patients; the balance between the volume and acuity of patients and the overall numbers of staff to attend them; and the balance between providing the service and training the workforce; and between the volume of senior clinical decision makers to the volume of junior clinical decision makers. Recent evidence from the UK resident doctors’ strike in which consultants undertook the resident doctors’ work reported that ED patients were seen, treated and discharged faster on strike days, despite fewer staff on duty, with no rise in deaths or re-admissions, although this was viewed as an unsustainable way to run the service [49]. We suggest that other dimensions could also be a consideration to balance, such as cost, and these require further exploration with other types of stakeholders such as commissioners of services. Some of these issues are being explored in the quantitative element of the parent study, aiming to provide practical advice for clinicians, managers and commissioners of services [16]. 

The study was undertaken at an extraordinarily turbulent period; when services were recovering from the impact of the Covid-19 pandemic, when there were some of the highest patient attendance rates on record in the NHS, when there were strikes by all groups of health professionals over pay and conditions, and when there was considerable public, political and policy attention to these types of new non-medical professional roles in the UK, and in particular that of PAs. In 2024 the government commissioned the independent Leng Review [23] to address ‘*concerns were raised about safety and lack of clarity of the roles*,* and about impact on training and employment of resident doctors*’p1 [44]. The Leng report concluded that there were no convincing reasons to abolish the PA role, but that attention should be paid to specifying the role boundaries, working within a clear team structure led by a doctor [47]. In July 2025, the government accepted the recommendations of the Independent Leng Review to limit the role of PAs in EDs [50]. All of the issues raised in the Leng Review [44] have resonance with the mixed views of impact reported in this study for *all* types of NMPs in skill mixed teams in ED/UTCs. From our analysis of participants’ views on an ideal skill mixed staffing in ED/UTCs we concluded that – when NMPs are employed - senior clinicians and managers should pay attention to issues of appropriate availability of supervision, balance of senior decision-makers to others, and appropriate deployment considerations.

### Strengths and limitations

A strength of the study was that it ensured breadth and diversity in participants through recruiting both patients and staff from different regions and services in England. A potential limitation was that as volunteer participants they may have had strongly held views, either against or for NMPs, that they wished included in the study i.e. they were engaged and opinionated on the subject although this could also be seen as a strength. As we have noted the study was undertaken in a particularly turbulent time post pandemic in the UK and for the health service. This may have had some impact on the study findings as there were many difficulties in recruiting staff and patients. We had difficulty in recruiting volunteer patients in every site and staff in some, resulting in uneven distribution between the sites. Despite our best efforts of reminders and repeated invitations there was a limit to this in order to not seem to be pressuring patients and staff (an ethical conduct consideration in recruitment). This may be seen as a limitation; however, we obtained a breadth of type of participant in all sites. The absence of perspectives from non-clinician managers could be viewed as a limitation, however, the focus on the perspective of those receiving and delivering care ensured proximity to the focus of the study.

The use of four researchers in data collection and then the wider research team, including a PPI co-researcher plus the three study advisory groups in the analysis, provides support for the credibility of the findings. The breadth of experience in the research team and the advisory groups regarding the involvement of NMPs in the health service was strength in ensuring rigor in analysis, synthesis, and interpretation. We have been transparent in providing detail of our methods and our backgrounds. While a qualitative study may be seen by some as a limitation, we have generalised from our findings at a theoretical level and offered ideas for further empirical study.

## Conclusions

This study offers insights into the perceived impact of including NMPs, such as ACPs and PAs, within the skill-mix of ED/UTC teams. We identified that there were mixed positive and challenging views, which seems to be an enduring theme in the research examining new and emerging roles in the health professions. We identified that most perspectives accepted NMPs (with that group’s variation) in the skill-mix but that the concept of balance was considered important for the optimal skill-mix within the ED/UTC staffing. We noted that participants when considering the current skill-mix which includes NMPs in all UTCs and many EDs were looking for multiple elements to be in equilibrium -: between patient volume and staff numbers; between senior clinical decision makers and junior clinical decision makers; and between provision of service and provision of training. We suggest that that managers and policy makers should pay attention at the local level to these issues when employing NMPs in their skill mix. The attention to the local level is one that we are exploring further in the quantitative elements of the parent study.

## Supplementary Information

Below is the link to the electronic supplementary material.

Supplementary Material 1: Additional file 1 Topic guides for patients and staff members

Supplementary Material 2: Additional file 2 Further verbatim exemplar quotations

## Data Availability

The datasets used and/or analysed in this study are available from Associate Professor Mary Halter on reasonable request.

## Abbreviations

ACP: Advanced clinical practitioner
ED-ACP: Emergency care—advanced clinical practitioner
ED/UTCs: Emergency departments and urgent care treatment centres
ENP: Emergency nurse practitioner
FTE: Full time equivalent
NHS: National Health Service
NMP: Non-medical practitioner
NPs: Nurse practitioners
PAs: Physician associates/assistants
PPI: Patient and public involvement group
RCEM: Royal College of Emergency Medicine
UK: United Kingdom
USA: United States
WHO: World Health Organisation
