# Supplementary material for: Perspectives on the impact of the inclusion of non-medical practitioners in the skill mixed staffing teams of English emergency departments: a qualitative study
Source: BMC Health Serv Res. 2026 May 29;26:1040. doi: 10.1186/s12913-026-14832-4 (PMC13430847; doi:10.1186/s12913-026-14832-4)
Supplement: Supplementary file 1 — Supplementary Material 1: Additional file 1 Topic guides for patients and staff members [file 12913_2026_14832_MOESM1_ESM.docx]

***Perspectives on the impact of the inclusion of non-medical practitioners in the skill mixed staffing teams of English emergency departments: a qualitative study* Additional File 1 Topic guides for patients and staff members**

**Implementation of the non-medical practitioner workforce into the emergency and urgent care system skill-mix in England: a mixed methods study of configurations and impact**


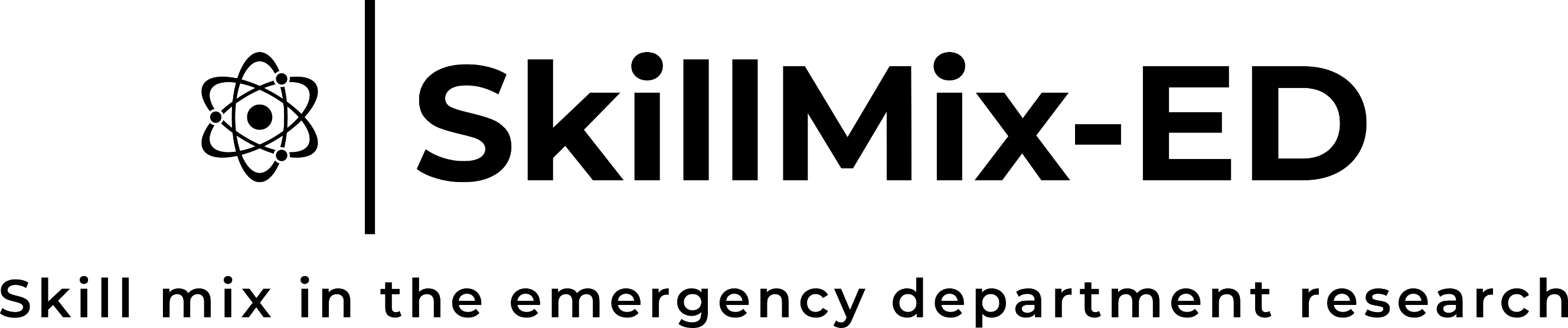


**INTERVIEW TOPIC GUIDE**

**for patients**

**Instructions for researcher**

The interviewees will have received full participant information previously. Confirm that the interviewee understands:

- the purpose of the research
- what the interview entails
- how confidentiality and anonymity will be assured
- that they have had chance to ask questions
- they are content to be recorded digitally or not
- they can stop at any time without explanation

Confirm the consent form is signed/returned.

Confirm with the participant at the start of the digital recording that they have consented to the recording.

In event of distress or disclosure please use – Standard Operating Procedure_WP5and WP6_Distress and Disclosure Instruction for Researchers_v1.0_2022 September 28^th^.

At the end thank them and ask if they wish to receive a summary of this part of the study, study updates, details of any journal articles published and/or a copy of the final report and summary. If yes, let them know we will send a separate email for their permission to place their contact details on a dissemination list until 15 months after the end of the study.

***Topic areas for open questions***

Interviewer to probe on all answers to ensure the meaning is clear (e.g. that is an interesting point, can you explain a bit more about it) and check for understanding (e.g. so can I check I have understood you correctly).

- Confirm the patient is/was a patient in the emergency department/urgent treatment centre
- Ask them if they recall which types of staff were in the staff team that attended them
- Ask them to describe the type of care they have been in receipt of without giving personal medical details
- Explore views on how the staff team members worked with each other e.g., inter-staff communication, teamwork with doctors, nurses, others
- Explore views on the skill mix in the staff team
- Explore views on communication, explanation, health education and discharge processes and which staff where involved in that
- Explore whether they felt treated safely and without harm
- Ask them how satisfied they were overall with their treatment
- Explore views on the outcome of their visit
- Ask them how they understand the role of the non-medical practitioner (provide examples of non-medical practitioners if needed) in the emergency department/urgent treatment centre
- If aware attended by a non-medical practitioner, ask if any examples of them working independently/with supervision
- If they were to need similar emergency care in the future, would they be content to receive similar care involving this type of staff team with a non-medical practitioner (and can they explain why) or would they prefer someone different? (And if yes, can they explain why?)
- Anything else they would like to say?

Ask participant if they are willing to provide their age, gender and ethnicity.

**Thank the participant**

**Confirm process of permissions for retention of contact details and for what types of information about the study.**

**Implementation of the non-medical practitioner workforce into the emergency and urgent care system skill-mix in England: a mixed methods study of configurations and impact**


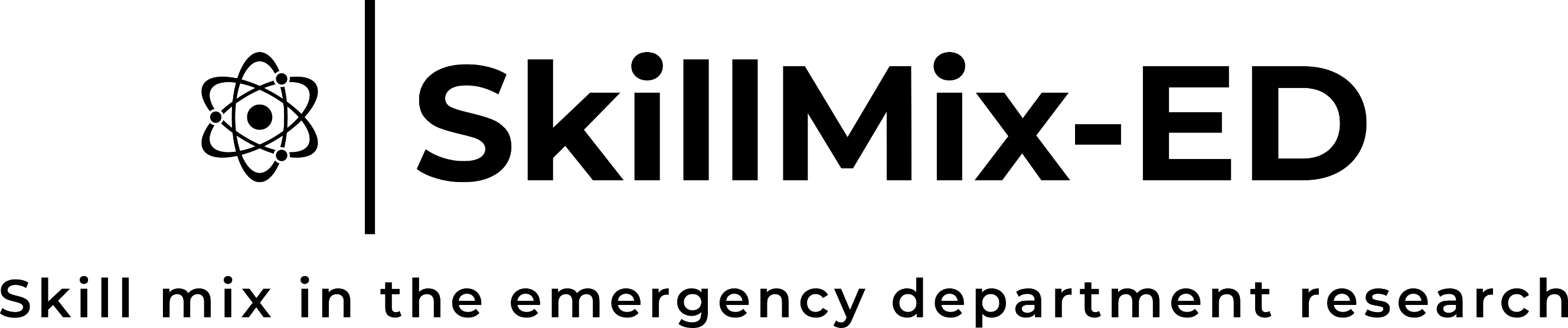


**INTERVIEW TOPIC GUIDE**

**for staff**

**Instructions for researcher**

The interviewees will have received full participant information previously. Confirm that the interviewee understands:

- the purpose of the research
- what the interview entails
- how confidentiality and anonymity will be assured
- that they have had chance to ask questions
- they are content to be recorded digitally or not
- they can stop at any time without explanation

Confirm the consent form is signed/returned.

Confirm with the participant at the start of the digital recording that they have consented to the recording.

In event of distress or disclosure please use– Standard Operating Procedure_WP5and WP6_Distress and Disclosure Instruction for Researchers_v1.0_2022 September 28th

At the end thank them and ask if they wish to receive a summary of this part of the study, study updates, details of any journal articles published and/or a copy of the final report and summary. If yes, let them know we will send a separate email for their permission to place their contact details on a dissemination list until 15 months after the end of the study.

***Topic areas for open questions***

- Confirm the participant works in the ED/UTC and clarify post, role and time/experience in ED/UTC
- Ask them to describe any experience working with non-medical practitioners/or as a non-medical practitioner in emergency services (check the range/role e.g., nurse practitioners, physician associates, paramedics, others e.g., pharmacists)
- Probe if any aspect unclear e.g., over what time period, type of involvement e.g. working

along side

- Explore participant’s views on how interdependently (how independently or with what level of supervision) non-medical practitioners/ or they as a non-medical practitioner work as part of the team and with senior staff in the ED/UTC
- Probe what works well and what works less well in this respect
- Explore the participant’s experience as to the impact or otherwise of non-medical practitioners/or their impact as a non-medical practitioner, in the team the ED/UTC
- Probe if any positive or negative impacts
- Probe what type, on who, from who/which perspective, on what aspect of the service delivery
- Explore the participant’s perspectives on the benefits and/or problems of non-medical practitioners in the skill-mix teams in the ED/UTC
- Probe perceived reasons for these
- Explore participant’s experience /views as to the right balance of skill mix in EDs and UTC
- Probe reasons for these views
- Explore participant’s views on where, if any, improvements could be made in relation to the implementation of non-medical practitioners in the skill-mix teams of EDs/UTCs
- Probe reasons for these views
- Explore participant’s views on what successful/or the ideal implementation of non-medical practitioners in the skill-mix teams of EDs/UTCs would look like
- Probe reasons for these views
- Anything else they would like to say?

**Thank the participant**

**Confirm process of permissions for retention of contact details and for what types of information about the study.**
