# Supplementary material for: Perspectives on the impact of the inclusion of non-medical practitioners in the skill mixed staffing teams of English emergency departments: a qualitative study
Source: BMC Health Serv Res. 2026 May 29;26:1040. doi: 10.1186/s12913-026-14832-4 (PMC13430847; doi:10.1186/s12913-026-14832-4)
Supplement: Supplementary file 2 — Supplementary Material 2: Additional file 2 Further verbatim exemplar quotations [file 12913_2026_14832_MOESM2_ESM.docx]

**Perspectives on the impact of the inclusion of non-medical practitioners in the skill mixed staffing teams of English emergency departments: a qualitative study**

**Additional file 2. Further verbatim exemplar quotations**

| **Theme and sub theme** | **Additional illustrative quote** |
| --- | --- |
| Positive contribution to dealing with high volumes of patients. | “Positive-wise, there are more people reviewing patients…In general, it's in the waiting times, so I would say the patients, it's good for the patients to have ANPs”. Nurse 2045 |
| Continuity in NMP employment in the ED contributing to efficiency and patient safety | “I like the fact that they [NMPs] all stay in the same place, unlike the junior doctors who move around. You have that continuity so from a patient safety perspective I really like that”. Emergency medicine speciality doctor 4688 |
| NMP’s viewed positively as adding value to clinical assessment and planning with patients | “ They [NMPs] can give you [the patient] advice, I very often hear them [ NMPs] saying, what to do to avoid things to get worse, what should change, they're asking about diet, they're asking about their lifestyle. So the patients need like that advice. The doctor, they will say your blood is fine, there's nothing on X-ray, goodbye; not all of them, but I know there is some like that because they don’t have the experience”. Nursing Associate 2270 |
| NMPs viewed as approachable by nurses and thought to positively add to team communication | “I have no problems going with my questions to a consultant or to a doctor or whatever, but if you have more junior staff like the newly qualified, they find that the ACPs are a little bit more approachable”. Senior nurse, 1962 |
| Problematic variation in NMPs role boundaries | “In terms of PAs, the fact they can't prescribe. Advanced Clinical Practitioners, obviously, can. All the ones that I work with can prescribe to a degree. The level of prescription required is where the holdup would be”. Patient also a NHS staff member 5383 |
| Potential negative impact on patient care of some NMPs not having legal authority to prescribe | “You probably know sepsis has to be treated quite quickly. Within an hour, you have to do certain things. If, let's say, there's no doctors - it does occasionally happen on night shifts, let's say, that a doctor will be working across a couple of areas while another one is on his break, and there's a PA left in the department with their patients. If a patient flags for sepsis, you would have to walk maybe five minutes down to the other side of the department, find the doctor, hopefully they're not with a patient, and things like that. If they [PAs] were able to prescribe, it would speed up care”. Senior nurse 6872 |
| Variation in NMPs’ seeking advice from doctors. | “If you're an ACP that's a little bit, shall we say, overconfident or even underconfident, this is what I've noticed, that can dictate that [oversight interaction with senior medical staff] quite heavily. Some ACPs will lean more on the senior doctors for support quite a lot. Others will - the other end of the spectrum, they will perhaps be a little bit - not know when to go and ask or should ask earlier”. NMP (nurse) 4038 |
| Positive views of the contribution of ENPs in UTCs | “I think we couldn't do our job or run the department without them [ENPs], I think they are essential and a core aspect of the UTC team” General practitioner in UTC 5051  “really appreciate the job that ENPs do. I really appreciate how well-scoped their role is, and in fact, I feel like they are probably better at seeing the cases they see than general doctors. For example, a fracture, they've seen thousands of fractures because that's the only thing they do, they see fractures, they see lacerations, they see injuries mostly, so they are very, very, very good at those, and I highly appreciate their jobs”. Junior fellow in emergency medicine 5270 |
| Challenging or negatives views of only the ENPs staffing of the minor injuries of the TUC area. | “the doctors don't go over there [Minors/UTC], the A&E doctors, then their skill is low, you know. They'll say, 'Nurse, this is going to take me forever because I don't know when last I've done a suture.' So, then those of us [nurses] who's qualified years ago, we will go ahead and do it.” Senior Nurse 2676 |
|  | “Since the introduction of ENPs who have taken care of the minor injuries……., us doctors don't get enough exposure in minor injuries. ……. newer doctors are not competent in minor injuries…. So previously, we used to see a couple of complex cases, and you could see one minor one just to have a variation of cases. But now the variation is becoming less and less, ….So that kind of affects, your day-to-day working, and sometimes can, I think, probably cause burnout as well”. Training registrar ST6. 1365 |
